# On an Inflammatory Fever Which Prevailed in the 24th Regiment in 1813

**Published:** 1814-10

**Authors:** G. F. Burroughs

**Affiliations:** Surgeon, *And late Assistant Surgeon of the 1st Dragoons*. 28, Park-street, Bristol


*For the Medical and Physical Journal.*

### *On an Inflammatory Fever which prevailed in the 24th Regiment in* 1813; *by* G. F. Burroughs, of Bristol.

OF all the diseases incident to the human frame, there are none so strongly characterised as those that prevail among soldiery at camps. How far this circumstance arises from the labour, fatigue, and irregular repose which soldiers on service undergo, I shall not here determine.

The disease which I am about to notice, prevailed to an alarming extent in the British army in Portugal, at the commencement of the year 1813, under the type of inflammatory fever. The summer preceding had been very fine; the troops had been constantly in motion from the month of May (at which period they broke up from their cantonments) to the middle of November. The halting places were in general good; and the men suffered no inconvenience from the night dews. The latter end of September, and the greater part of the month of October, was very cold and rainy; and the right wing of the army was vigorously employed in besieging Burgos. The duties of the soldier were at this time very severe, and particularly of those employed in the trenches.

trenches. Towards the latter end of October, the troops broke up from Burgos, and retreated to Portugal. In this retreat it was impossible to avoid feeling all those privations which retreating armies are necessarily exposed to—want of due rest, scarcity of provisions, and, above all, wet grounds (from the inclement and unfavourable state of the weather) for *bevouac*, so that the troops were scarcely ever dry.

It was not until the beginning of December that the troops occupied their respective cantonments, part remaining on the south bank of the river Douro, and part the north-west side of the river Coa. On their gaining these stations, the troops were regularly supplied with their usual allowances of bread, meat, and wine. My own battalion occupied a village on the western side of the Estrella mountains, by which it was sheltered from the bleak cold winds that are apt to blow for a long time from the east at this season of the year. Its situation was healthy, and a limpid stream ran through it. We were not here many days before the sick list was considerably increased, for hitherto the regiment had been very healthy, indeed as long as the men were actively employed it suspended disease.

The men were taken sick generally when on duty, and were brought to the hospital labouring under these symptoms:—cold rigors, with alternate flushes of heat; pains in the forehead, back, and lower extremities; vomiting of yellow, and often inspissated bile, now and then accompanied with the discharge of long and round worms; pulse quick; tongue furred, though sometimes clean; and great irritability of the pupils. These symptoms were not unfrequently attended by a short and dry cough, with sensations of pain referred to particular parts of the thorax or abdomen.

Of the treatment of this disease, which I have before observed was of an inflammatory kind, not a doubt could be entertained; but the repeated fatigues our soldiers had undergone had reduced them to a very emaciated state, and we could not well ascertain how far the antiphlogistic treatment could be carried. Emetics, the common routine practice of the army, were primarily administered, but their operation so powerfully disturbed the sensorium, that they were afterwards forbidden,—indeed we found our patients in general much worse after their use. Purgatives, particularly calomel combined with the extract of colocynth, were administered in moderate doses with considerable effect.

Towards evening the symptoms were uniformly increased. The skin became hot, with no disposition to moisture; respiration laborious; and pain in the head acute: there was also also a bad taste in the mouth, and the tongue was white, with very red papillæ. Even in the patient's countenance there was a wildness, bordering on delirium, and an unusual rapidity of utterance. Venesection was instantly enforced, and the blood was drawn from large orifices, and in pretty large quantities, the pulse often rising under the operation with a fullness and hardness truly formidable. The blood drawn did not uniformly put on the buffy coat, but contained much crassamentum, with a smaller quantity of serum than usual.

On the second day, the symptoms continuing, we found it necessary to repeat the venesection twice, sometimes thrice, in the course of the twenty-four hours, and to assist the operation of the purgative, by the administration of an enema. On the third day, if there was no sign of amendment, if the evacuations both by the bowels and blood-letting had not contributed to arrest the phlogistic diathesis, if, with increased heat, the skin retained its rough and dry feel, then delirium intervened, and the patient expired under the most accumulated suffering.* But it was not an unfrequent occurrence, that in the course of twelve or sixteen hours from the first attack, a copious perspiration would break out on the whole surface of the body, and continue some hours, during which time the blankets would be completely moistened with the secreted matter.

This inflammatory fever ran through half the 24th regiment, and there was no doubt of its being contagious from the circumstance of the orderlies, or soldiers who attended on the sick in hospital, receiving the disease. These were generally seized with vertigo, shivering succeeded by vomiting, either on the first or second day; and so great was the dread the soldiers entertained of the unhealthiness of the hospital, that it was only through coercion they were induced to attend it. The women who were employed in washing the linen for the hospital, received the disease, and one of them died.

Of one hundred and sixty-five cases that were treated by blood-letting, and according to the antiphlogistic system, six only terminated fatally, and that at the commencement of the disease. The dissection of these cases gave us very satisfactory information, inasmuch as we found either inflammation of the pleura, with adhesion of the lungs to the thorax, or of the peritoneal coat of the stomach, and small

* In other instances, where the symptoms did not exhibit themselves so strongly, and where debility was very predominent, life was protracted to the seventh or tenth day.

intestines, intestines. The liver and spleen were generally found enlarged, and not unfrequently scirrhus. Though these morbid appearances presented themselves on viewing the cavities of the thorax and abdomen, there were no less formidable ones on the external parts of the body; and such was the impoverished state of these subjects, that gangrene of the lower extremities, and lividness of the tip of the nose, had commenced at the very time they were labouring under visceral inflammation, so unequally were the powers of life distributed.

Whether this disease owed its origin to the affection of any individual organ, I am not well satisfied; certain it is, the symptoms of local inflammation were not decided, at its first appearance, but afterwards we found either the thoracic or abdominal viscera partaking highly of inflammatory action, for which, with other depleting remedies, blistering the affected part was very serviceable.

It is a fact worth recording, that of those men who were bled, the orifices made in their arms never kindly healed, until the inflammatory disposition of the system had disappeared. The edges of the wound became inflamed and hard, sometimes even affecting the glands in the axilla.

In some constitutions, particularly the robust, the fever was always more violent; and the passage of the urine along the urethra was attended with the most indescribable anguish.

I have observed that the blood drawn from the patients did not uniformly cup, and put on the buff colour, but in most cases it did, and at the very time it was flowing from the arm.*

Bleeding was so important a remedy, and the disease was so early arrested by its vigorous and timely enforcement, that our practice bore a strong analogy to the observation made by Sir John Pringle, when speaking of the disorders that prevailed in Flanders in the winter of 1745, and which were also of the inflammatory kind. "The physician of the army," he says, "believed the surgeons and apothecaries of the place more than half instructed about the cure of the patients committed to them, when he had inculcated the necessity of large and repeated bleedings."

The subjects of this fever, (which may be regarded as the synocha of Cullen) either by its ravages, by the remedies employed, or from previous fatigue, or perhaps from the

* The subsidence of the blood is by no means an invariable criterion of the existence of inflammation; and, where there were evident pulmonic symptoms, the blood coagulated, as when in a healthy state.

operation operation of all these cases, were uniformly left in a very exhausted condition, and required the greatest attention both to their regimen and exercise, during convalescence; and to this attention we had to congratulate ourselves that our relapses were but few, and yielded to gentle treatment.

"Inflammatory fevers of an army, differ from others only in being more violent," observes Sir John Pringle, and perhaps it is only in an army actively employed, that diseases assume the more formidable aspect. The complaint I have detailed was of this nature, and demanded prompt and decisive practice. It prevailed through the whole of the army, and the mortality was greater than the loss we had experienced the preceding summer, in the battle of Salamanca, and siege of Burgos.

In those corps where venesection was delayed, or only little resorted to, the mortality was very great; but in those who employed it early, the mortality was inconsiderable.

G. F. BURROUGHS, Surgeon,

*And late Assistant Surgeon of the 1st Dragoons.*

28, *Park-street, Bristol.*

